# The Role of *FpfetC* from *Fusarium proliferatum* in Iron Acquisition, Fumonisin B1 Production, and Virulence

**DOI:** 10.3390/ijms26072883

**Published:** 2025-03-22

**Authors:** Ling Wang, Wen Li, Shuailing Ge, Zhonghua Sheng, Shikai Hu, Guiai Jiao, Gaoneng Shao, Lihong Xie, Shaoqing Tang, Peisong Hu

**Affiliations:** State Key Laboratory of Rice Biology and Breeding, China National Center for Rice Improvement, China National Rice Research Institute, Hangzhou 311401, China; lw159267@163.com (W.L.); wsgsldyx@163.com (S.G.); shengzhonghua@caas.cn (Z.S.); hushikai@caas.cn (S.H.); jiaoguiai@caas.cn (G.J.); shaogaoneng@caas.cn (G.S.); xielihong@caas.cn (L.X.); tangshaoqing@caas.cn (S.T.)

**Keywords:** *Fusarium proliferatum*, multicopper ferroxidase, iron acquisition, fumonisins, virulence

## Abstract

Iron is an essential micronutrient required for the fungal growth and propagation. *Fusarium proliferatum* is the causal agent of rice spikelet rot disease. In this study, we characterized the role of *F. proliferatum* multicopper ferroxidase (FpfetC), which mediated the oxidization of ferrous to ferric iron in the reductive system of iron assimilation. Deletion of *FpfetC* led to impaired growth under iron-deprived conditions, and the growth defect could be restored by exogenous iron. Compared to wild-type Fp9 strain, Δ*FpfetC* showed increased conidiation, resistance to copper stress, and sensitivity to zinc stress. *FpfetC* deficiency rendered a transcription remodeling of genes involved in high-affinity iron assimilation, iron homeostasis and iron storage. Moreover, production of fumonisin B1 (FB1) and transcript levels of fumonisin biosynthesis (*Fpfums*) genes were elevated in Δ*FpfetC*. Δ*FpfetC* exhibited hypervirulence to rice, accompanied with aggravation of invasive hyphae and activation of siderophore synthesis at the sites of inoculation. Additionally, disruption of *FpfetC* attenuated penetration ability to cellophane membrane under iron starvation. Taken together, these results demonstrated that *FpfetC* played important roles in iron uptake, conidiation, response to metal stress, fumonisin biosynthesis, and virulence in *F. proliferatum*.

## 1. Introduction

Iron is an essential trace element in living organisms. It serves as a catalytic cofactor for many enzymes and regulatory proteins in a series of physiological processes, such as mitochondrial respiration, DNA replication and repair, synthesis of amino acids, tricarboxylic acid cycle, and lipid and sterol metabolism [1]. However, iron overload is detrimental to cells because reactive oxygen species are generated via Haber-Weiss/Fenton reaction [2]. Iron homeostasis needs to be exquisitely manipulated to balance acquisition, storage, and consumption of iron. To overcome iron deficiency, the fungi have evolved high-affinity acquisition systems, termed as siderophore-mediated iron acquisition (SIA) and reductive iron assimilation (RIA) [3]. The RIA pathway is started with extracellular reduction in ferric iron (Fe^3+^) by ferric reductase (Fre family) into ferrous iron (Fe^2+^), which was re-oxidized into Fe^3+^ iron by multicopper ferroxidase (Fet family), subsequently being transported into the cytosol by ferric permease (Ftr family) [4]. The RIA system played a pivotal role in the fungal pathogenesis. In the opportunistic pathogen *Cryptococcus neoformans*, overexpression of *Fre3* gene increased the infection ability during the environment-to-mammal niche transition [5]. In the hemibiotrophic fungus *Colletotrichum graminicola*, deletion of *Fet3-1* gene led to a decline in appressorium penetration, biotrophic development, and the formation of necrotic lesions on maize [6]. In the entomopathogenic fungus *Beauveria bassiana*, Fre and Ftr proteins contributed to iron-dependent growth, blastospore development, and fungal virulence [7,8]. Moreover, RIA was also known to be involved in melanin production, resistance against azole drugs, tolerance of oxidative stress, and laccase activity [9,10,11].

The filamentous ascomycete *Fusarium proliferatum* could infect a wide range of crops, such as rice, maize, wheat, barley, sorghum, asparagus, onion, and sugarcane [12,13]. Moreover, the fungus produces multiple mycotoxins, including fumonisins, fusaric acid, fusarins, moniliformin, and bikaverin [14]. Amongst them, fumonisins are of high concern as a group of notorious mycotoxins. So far, at least 28 fumonisin analogs have been identified and designated as A, B, C, and P series [15], with fumonisin B1 (FB1) being the most common and toxic [16]. Exposure to FB1 is linked to acute and chronic toxicity in livestock, such as leukoencephalomalacia in equids, pulmonary edema in swine, and liver cancer in rats, as well as esophageal cancer and neural tube defects in humans [17]. FB1 has been classified as a possible carcinogen to humans (group 2B) by the International Agency for Research on Cancer (IARC) [18]. Maximum permissible levels for FB1 in foods and feedstuff have been set by the European Union, United States Food and Drug Administration, and Codex Alimentarius Commission [19]. To ensure food security and human health, the effective approaches should be performed to eliminate and/or mitigate fumonisins contamination in agro-ecosystems.

Rice (*Oryza sativa* L.) is the primary cereal crop, feeding more than half of the world’s population [20]. Being the largest producer and consumer in the world, China has approximately 30 million hectares of rice cultivation area [21]. Rice spikelet rot disease, predominantly caused by *F. proliferatum*, is one of the most prevalent diseases in China [13,22]. Over the past few decades, the disease has become continuously widespread, particularly in the warm and humid regions along the middle-and-lower reaches of the Yangtze River Delta [14]. Annually, the occurrence of the disease covers nearly 800,000 hectares in China [14]. When the rice booting and heading stages encounter consecutive rainfall events, the incidence of diseased spikelets can reach up to 80% in paddy fields [13]. Epidemics of rice spikelet rot not only resulted in yield losses but also led to mycotoxin contaminants in grains. However, disease management remains challenging, owing to lack of effective fungicides and resistant sources [13]. Insights into the pathogenicity mechanisms of *F. proliferatum* are crucial for the development of control strategies. To date, little is known about iron uptake and homoeostasis or its relevance to virulence in *F. proliferatum*. Here, the objectives of the present study were (1) to determine the effect of *FpfetC* gene encoding multicopper ferroxidase of *F. proliferatum* on vegetative growth and conidiation, (2) to clarify the potential influence of *FpfetC* on iron-dependent processes, (3) to evaluate whether *FpfetC* participated in the stress response to metal ions, (4) to identify the role of *FpfetC* in fumonisins biosynthesis, and (5) to verify the contribution of *FpfetC* to pathogenicity.

## 2. Results

### 2.1. Identification and Expression of FpfetC in F. proliferatum

**Figure 1:**
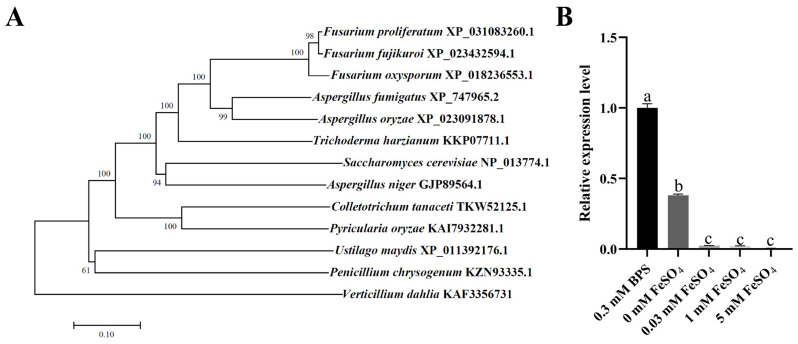
Identification and expression of multicopper ferroxidase (FpfetC) in *F. proliferatum*. (**A**) Phylogenetic relationship of FpfetC protein and its homologs from different filamentous fungi. A phylogenetic tree was constructed by neighbor-joining method with 1000 bootstrap replicates using MEGA11 software (version 11.0). Bootstrap values were denoted at the supported node. The species names and GenBank accession numbers of the organisms were shown within the clade. (**B**) Expression of *FpfetC* gene under different concentrations of iron. The Fp9 strain was inoculated in MM media omitting iron at 28 °C for 3 days, then shifted into MM media supplemented with different concentrations of iron for 2 h. Expression value of *FpfetC* gene in Fp9 strain grown in MM media with BPS was artificially set as 1. Data are presented as mean ± standard deviation. Error bars denote standard deviation from three biological replicates. Different lowercase letters indicate significant differences as determined using ANOVA followed by Student’s *t*-tests at *p* < 0.05. There were three replicates for each sample. The experiment was repeated three times.

Employing the amino acid sequence of multicopper oxidase Fet3 from *Saccharomyces cerevisiae* as a query, one putative homologue was retrieved from *F. proliferatum* by BLASTP analysis, designated as FpfetC. The protein sequence of FpfetC shared 47% identity with Fet3 sequence of *S. cerevisiae*. Domain analysis showed that FpfetC contained three cupredoxin-like domains. Phylogenetic analysis indicated that fetC protein was highly conserved in filamentous fungi, and FpfetC had the closest relationship with orthologs from other *Fusarium* species (Figure 1A).

The mRNA levels of *FpfetC* were obviously increased in the presence of iron chelator bathophenanthroline disulfonate (BPS) or no iron, but were decreased in minimal medium (MM) with addition of iron (0.03 mM, 1 mM, and 5 mM FeSO_4_) (Figure 1B). The results indicated that expression of *FpfetC* gene was induced under iron-limited conditions.

To gain insights into the biological functions of *FpfetC* in *F. proliferatum*, the deletion mutant Δ*FpfetC* was constructed by targeted replacement of *FpfetC* gene with hygromycin (*HYG*) resistance cassette in Fp9 strain (Figure S1). PCR and Southern blotting analysis confirmed the site-directed insertion with single copy. Reintroduction of an intact copy of *FpfetC* gene into the Δ*FpfetC* mutant generated the complementary strain Δ*FpfetC*-C (Figure S2).

### 2.2. Loss of FpfetC Decreased Growth Under Iron-Limited Conditions

**Figure 2:**
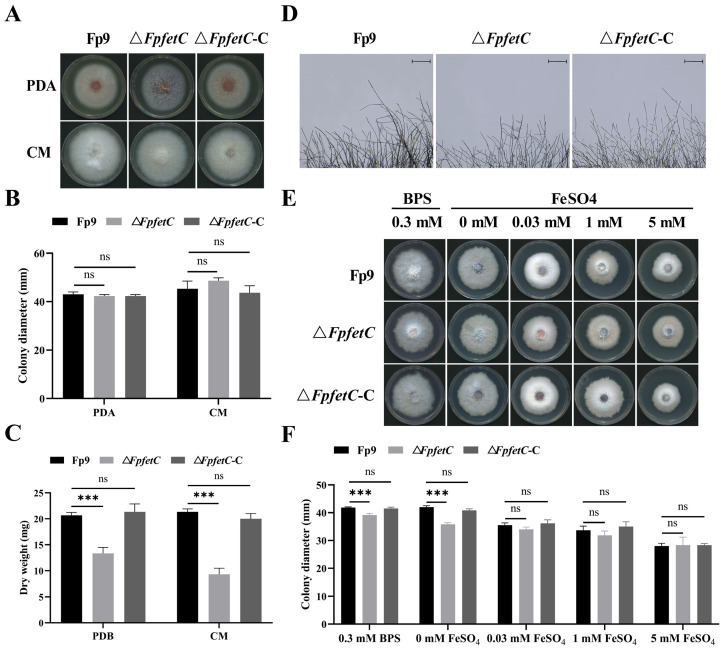
Effect of *FpfetC* on colony growth in *F. proliferatum*. (**A**) Colony morphology of Δ*FpfetC* cultured on PDA and CM media at 28 °C for 5 days. (**B**) Colony diameter of Δ*FpfetC* on PDA and CM media at 28 °C for 5 days. (**C**) Mycelial biomass of Δ*FpfetC* inocubated in PDB and CM liquid media at 28 °C for 4 days. (**D**) Hyphal tips of Δ*FpfetC* grown on PDA media at 28 °C for 36 h. Scale bars, 500 μm. (**E**) Colony morphology of Δ*FpfetC* cultured on MM media supplemented with different concentrations of iron at 28 °C for 5 days. (**F**) Colony diameter of Δ*FpfetC* cultured on MM media supplemented with different concentrations of iron at 28 °C for 5 days. Data are presented as mean ± standard deviation. Error bars denote standard deviation from three biological replicates. Asterisks indicate statistical significance as determined using ANOVA followed by Student’s *t*-tests (ns—not significant; ***—*p* < 0.001). Each experiment was carried out with three replicates and performed three times.

No significant difference was observed in colony growth between Δ*FpfetC* and the Fp9 strain on nutrient-rich media, including potato dextrose agar (PDA) and complete medium (CM) media (Figure 2A,B). Relative to the Fp9 strain, the mycelia biomass of Δ*FpfetC* was reduced in liquid cultures (Figure 2C) and aerial hyphae appeared more sparse and rarely branched (Figure 2D).

To determine whether colony growth depended on iron availability, the strains were grown on MM media supplemented with various concentrations of iron. The colony growth of Δ*FpfetC* was decreased under iron-deprived conditions (0.3 mM BPS and no iron) compared to that of the Fp9 strain (Figure 2E,F). After adding exogenous iron (0.03 mM, 1 mM or 5 mM FeSO_4_) into the MM media, the growth defect of Δ*FpfetC* was restored. The colony phenotype of Δ*FpfetC*-C was similar to the Fp9 strain at each iron concentration. The observations reflected that *FpfetC* was required for colony growth when iron was depleted in *F. proliferatum*.

### 2.3. Deletion of FpfetC Stimulated Formation of Asexual Conidia

**Figure 3:**
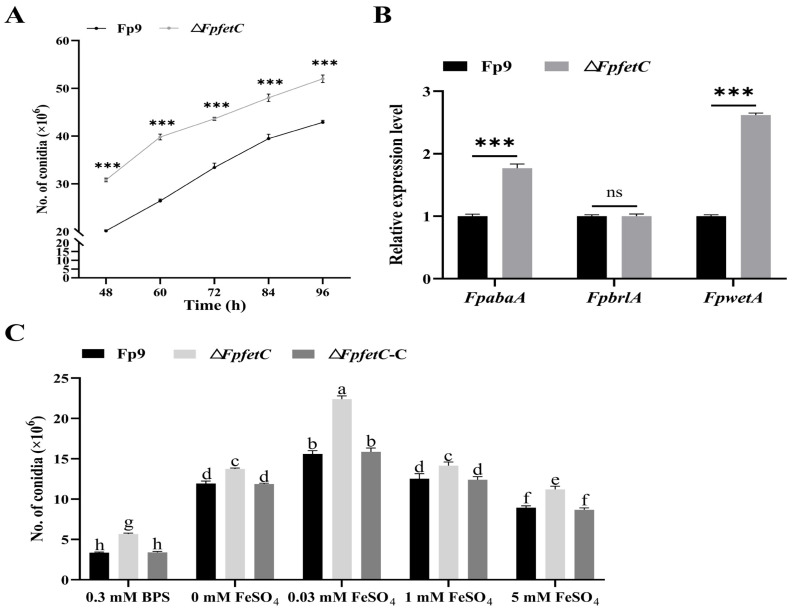
Impact of *FpfetC* on conidiation in *F. proliferatum*. (**A**) The amount of conidia of Δ*FpfetC* cultured in YEPD media at 28 °C. The sporulation was recorded at intervals of 12 h. (**B**) Relative expression levels of conidiation-related genes *FpabaA*, *FpbrlA*, and *FpwetA*. Expression value of each gene in Fp9 strain grown in YEPD media was artificially set as 1. (**C**) The amount of conidia of Δ*FpfetC* under different concentrations of iron. After culturing in MM media omitting iron at 28 °C for 3 days, Δ*FpfetC* was transferred into MM media supplemented with different concentrations of iron for 4 days. Data are presented as mean ± standard deviation. Error bars denote standard deviation from three biological replicates. Asterisks indicate statistical significance as determined using ANOVA followed by Student’s *t*-tests (ns—not significant; ***—*p* < 0.001). Different lowercase letters indicate significant differences at *p* < 0.05. Each experiment was carried out with three replicates and performed three times.

There was a greater abundance of conidia of the Δ*FpfetC* mutant compared to the Fp9 strain in yeast extract peptone dextrose (YEPD) media (Figure 3A). Meanwhile, expression of two transcription factors associated with conidiation, namely *FpabaA* and *FpwetA*, was markedly upregulated in Δ*FpfetC* (Figure 3B).

To elucidate the influence of iron levels on conidiation, the strains were grown in MM liquid media containing different concentrations of iron. The amount of conidia of Δ*FpfetC* was higher than that of the Fp9 strain during both iron-deprived and iron-replete conditions (Figure 3C). For each strain, the number of conidia was the lowest in the treatment of BPS, gradually rose and peaked in MM media supplemented with 0.03 mM FeSO_4_, then declined with the increment of iron concentration. Δ*FpfetC*-C displayed the same amount of conidia as the Fp9 strain at any iron concentration. Collectively, these data underlined that *FpfetC* was involved in asexual sporulation, regardless of the iron levels.

### 2.4. Deletion of FpfetC Altered Transcription Pattern of Iron-Associated Genes

**Figure 4:**
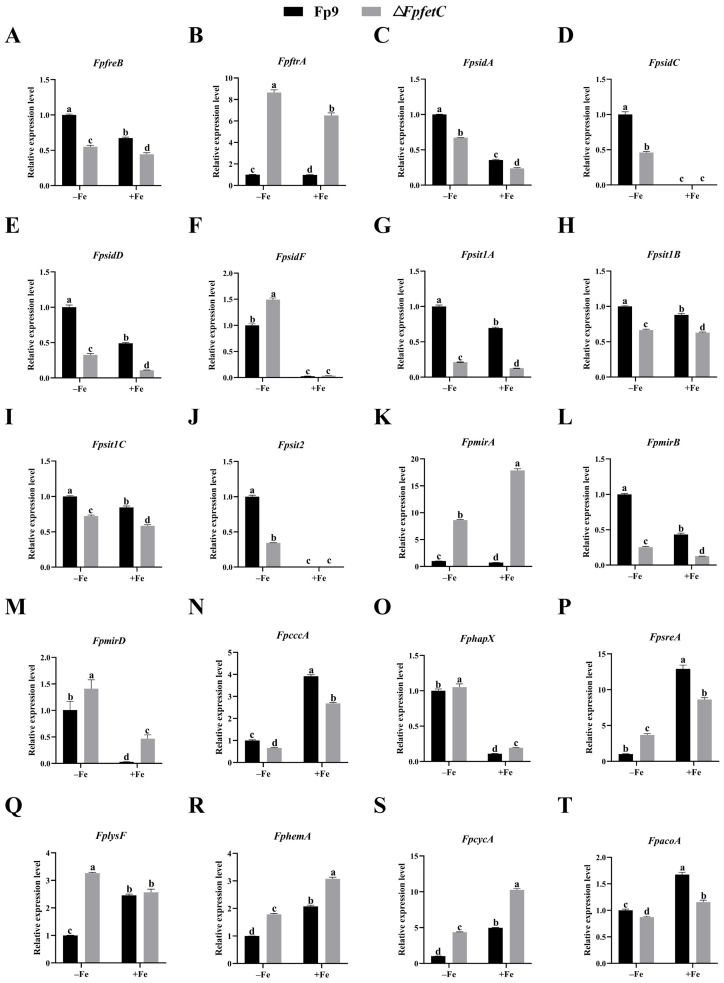
Involvement of *FpfetC* in expression of genes associated with iron metabolism. After preculturing in MM media omitting iron for 3 days at 28 °C, Δ*FpfetC* was transferred into MM media supplemented with 0.3 mM BPS (iron starvation, –Fe) or 0.03 mM FeSO_4_ (iron sufficiency, +Fe) for 2 h. Investigated genes were as follows: (**A**) *FpfreB* gene encoding ferric reductase and (**B**) *FpftrA* gene encoding iron permease, which were involved in reductive iron assimilation; (**C**) *FpsidA* gene encoding ornithine-N^5^-oxygenase, (**D**) *FpsidC* and (**E**) *FpsidD* genes encoding non-ribosomal peptide synthetases (NRPS), and (**F**) *FpsidF* gene encoding N^5^-transacylase, which were involved in siderophore biosynthesis; (**G**) *Fpsit1A*, (**H**) *Fpsit1B*, (**I**) *Fpsit1C* and (**J**) *Fpsit2* genes encoding siderochrome-iron transporters, which were involved in ferrichrome-type siderophore transport; (**K**) *FpmirA* gene encoding enterobactin transporter, (**L**) *FpmirB* gene encoding TAFC importer and (**M**) *FpmirD* gene encoding fusarinine C transporter, which were involved in fusarinine-type siderophore transport; (**N**) *FpcccA* gene encoding vacuolar iron importer, which was involved in iron storage; (**O**) *FphapX* gene encoding bZIP type transcription factor and (**P**) *FpsreA* gene encoding GATA type transcription factor, which were iron regulators; (**Q**) *FplysF* gene encoding homoaconitase, (**R**) *FphemA* gene encoding 5-aminolevulinate synthase, (**S**) *FpcycA* gene encoding cytochrome C, and (**T**) *FpacoA* gene encoding aconitate hydratase, which were involved in iron consuming. Expression value of each gene in Fp9 strain in MM media with BPS was artificially set as 1. Data are presented as mean ± standard deviation. Error bars denote standard deviation from three biological replicates. Different lowercase letters indicate significant differences as determined using ANOVA followed by Student’s *t*-tests at *p* < 0.05. There were three replicates for each sample. The experiment was repeated three times.

The levels of mRNA from genes responsible for reductive iron uptake (*FpfreB*, *FpftrA*), siderophore biosynthesis (*FpsidA*, *FpsidC*, *FpsidD* and *FpsidF)*, ferrichrome-type siderophore transport (*Fpsit1A*, *Fpsit1B*, *Fpsit1C* and *Fpsit2*), fusarinine-type siderophore transport (*FpmirA*, *FpmirB* and *FpmirD*), and transcription factor (*FphapX)*, were markedly higher under iron starvation than iron sufficiency (Figure 4). Conversely, the levels of mRNA from genes associated with iron consuming (*FplysF*, *FphemA*, *FpcycA* and *FpacoA*), iron storage (*FpcccA*), and transcription factor (*FpsreA)*, were strongly repressed under iron starvation (Figure 4). The findings indicated that transcription of iron-dependent genes was affected by iron starvation.

The influence of *FpfetC* on iron-associated genes was assessed in the presence or absence of iron. Deletion of *FpfetC* resulted in changes in the expression of most genes under both iron starvation and iron sufficiency (Figure 4). In the RIA system, the *FpfreB* gene was downregulated, but the *FpftrA* gene was upregulated in Δ*FpfetC*. Regarding siderophore biosynthesis and uptake, the transcripts of *FpsidA*, *FpsidD*, *Fpsit1A*, *Fpsit1B*, *Fpsit1C*, *Fpsit2*, and *FpmirB* genes were markedly reduced, while those of *FpmirA* and *FpmirD* genes were elevated in Δ*FpfetC*. For iron storage, expression of the *FpcccA* gene was downregulated in Δ*FpfetC*. With respect to iron consuming, transcript levels of *FphemA* and *FpcycA* genes were increased, whereas that of the *FpacoA* gene was decreased in Δ*FpfetC*. For iron homeostasis, mRNA levels of the *FphapX* gene under iron starvation and the *FpsreA* gene under iron sufficiency were decreased in Δ*FpfetC*. Together, these results demonstrated that *FpfetC* affected the transcription regulation of genes associated with iron metabolism in *F. proliferatum*.

### 2.5. Loss of FpfetC Affected the Response to Metal Ion Stresses

**Figure 5:**
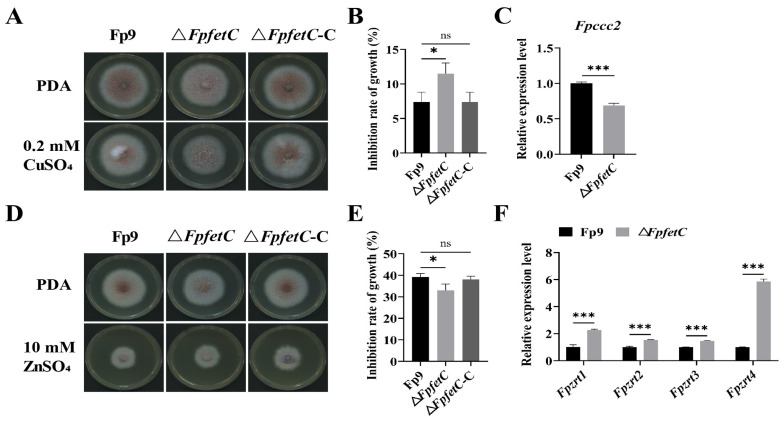
Role of *FpfetC* on sensitivity to the excesses of copper or zinc in *F. proliferatum*. (**A**) Colony morphology of Δ*FpfetC* grown on PDA media with or without 0.2 mM CuSO_4_ at 28 °C for 5 days. (**B**) Inhibition rate of mycelial growth of Δ*FpfetC* on PDA media with 0.2 mM CuSO_4_. (**C**) Relative expression level of *Fpccc2* gene encoding copper transport ATPase. After preculturing in PDB media for 48 h, Δ*FpfetC* was transferred to PDB media with 0.2 mM CuSO_4_ for 24 h. Expression value of *Fpccc2* gene in Fp9 strain was artificially set as 1. (**D**) Colony morphology of Δ*FpfetC* grown on PDA media with or without 10 mM ZnSO_4_ at 28 °C for 5 days. (**E**) Inhibition rate of mycelial growth of Δ*FpfetC* on PDA media with 10 mM ZnSO_4_. (**F**) Relative expression levels of *Fpzrts* genes encoding zinc-regulated transporter. After preculturing in PDB media for 48 h, Δ*FpfetC* was transferred to PDB media with 10 mM ZnSO_4_ for 24 h. Expression values of *Fpzrts* genes in Fp9 strain were artificially set as 1. Data are presented as mean ± standard deviation. Error bars denote the standard deviation from three biological replicates. Asterisks indicate statistical significance as determined using ANOVA followed by Student’s *t*-tests (ns—not significant; *—*p* < 0.05; ***—*p* < 0.001). Each experiment was carried out with three replicates.

When treated with 0.2 mM CuSO_4_, Δ*FpfetC* showed enhanced tolerance compared to Fp9 strain (Figure 5A,B), and transcript level of *Fpccc2* gene encoding copper transport ATPase was significantly reduced in Δ*FpfetC* (Figure 5C). After being exposed to 10 mM ZnSO_4_, Δ*FpfetC* was more sensitive than Fp9 strain (Figure 5D,E); meanwhile, expressions of zinc-regulated transporters (*Fpzrts*) were highly induced in Δ*FpfetC* (Figure 5F). No significant differences in phenotype were observed between Δ*FpfetC*-C and Fp9 strain under excesses of copper or zinc. Collectively, these results suggested that *FpfetC* was involved in sensitivity to copper and zinc stresses.

### 2.6. The Absence of FpfetC Led to Elevated Production of Fumonisin FB1

**Figure 6:**
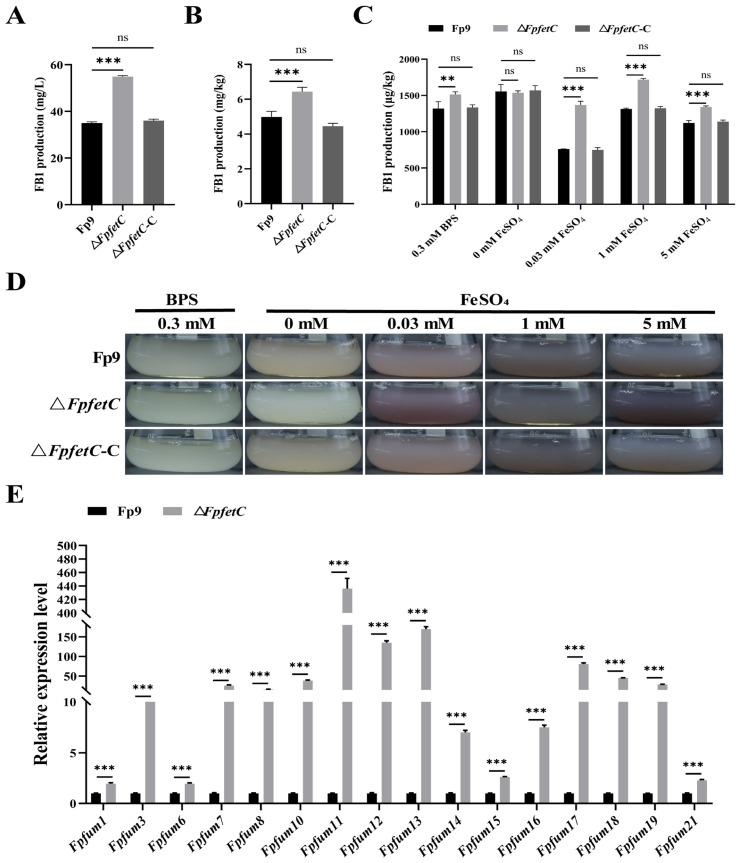
Effect of *FpfetC* on FB1 biosynthesis in *F. proliferatum.* (**A**) FB1 content produced by Δ*FpfetC* cultured in PDB media for 9 days. (**B**) FB1 content produced by Δ*FpfetC* cultured on cracked rice kernels for 14 days. (**C**) FB1 content produced by Δ*FpfetC* under different concentrations of iron. After being grown in MM media omitting iron at 28 °C for 4 days, Δ*FpfetC* was transferred into MM media supplemented with different concentrations of iron at 28 °C for 5 days. (**D**) Culture of Δ*FpfetC* grown in MM media with different concentrations of iron at 28 °C for 5 days. (**E**) Relative expression levels of *Fpfum* genes responsible for fumonisin biosynthesis. Expression value of each gene in Fp9 strain was artificially set as 1. Data are presented as mean ± standard deviation. Error bars denote standard deviation from three biological replicates. Asterisks indicate statistical significance as determined using ANOVA followed by Student’s *t*-tests (ns—not significant; **—*p* < 0.01; ***—*p* < 0.001). Three replicates were used for each sample, and the experiment was performed three times.

Deletion of *FpfetC* caused a much larger amount of FB1 than Fp9 strain, both in potato dextrose broth (PDB) media (Figure 6A) and on cracked rice kernels (Figure 6B). In terms of the impact of *FpfetC* on FB1 production under varied concentrations of iron, FB1 content in Δ*FpfetC* mutant was more than that in Fp9 strain at the identical concentrations of iron, except for iron-omitting condition (Figure 6C,D). In comparison, the highest level of FB1 produced by Δ*FpfetC* occurred at concentration of 1 mM FeSO_4_. Correspondingly, levels of mRNA from fumonisin biosynthetic (*Fpfum*) genes were increased in Δ*FpfetC* (Figure 6E). There was no obvious difference in FB1 content between Δ*FpfetC-C* and Fp9 strain under any culture condition. Overall, the results suggested that *FpfetC* negatively regulated FB1 production, which was independent of environmental iron status.

### 2.7. Loss of FpfetC Enhanced the Ability of Fungal Infection and Colonization

**Figure 7:**
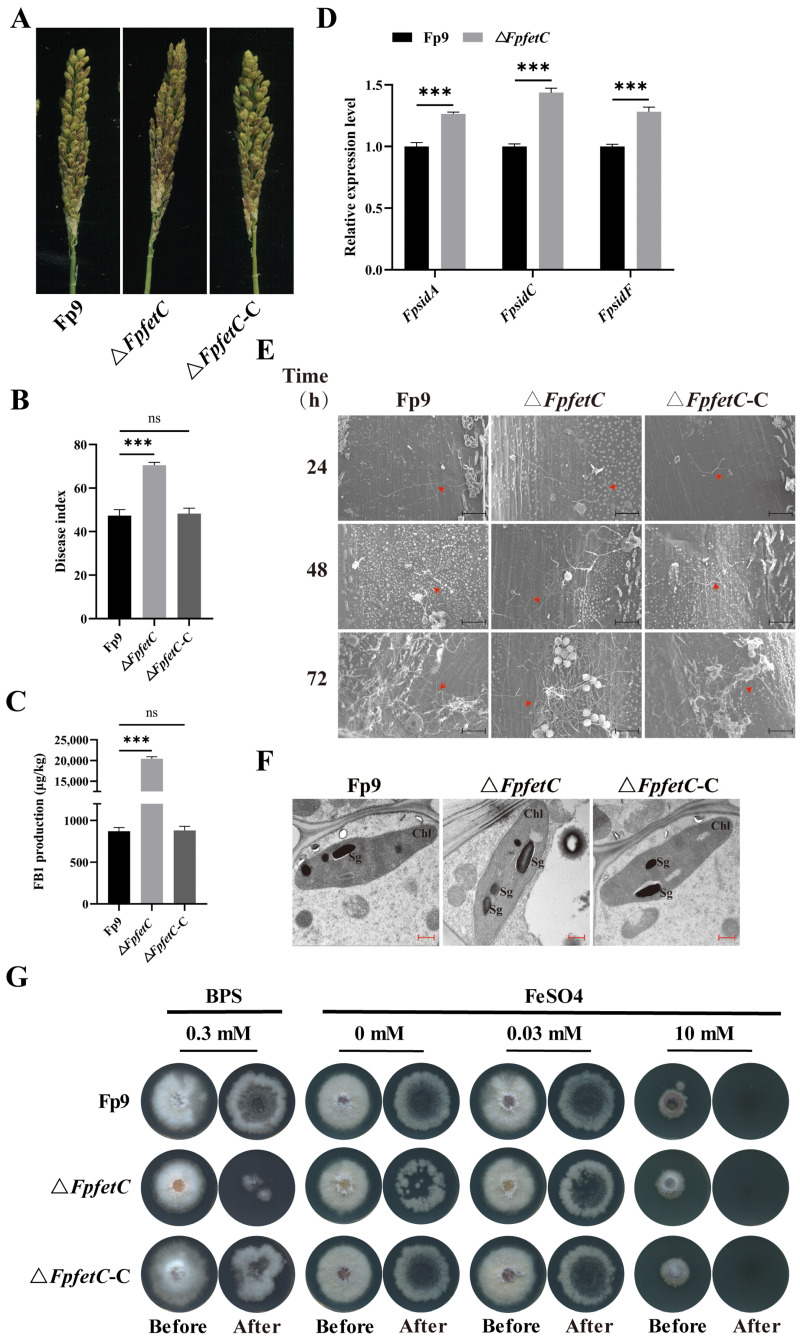
Influence of *FpfetC* on virulence in *F. proliferatum*. (**A**) Disease symptom of rice spikelets inoculated with Δ*FpfetC* at 21 days post-infection (dpi). (**B**) Disease index of rice spikelets inoculated with Δ*FpfetC* at 21 dpi. (**C**) FB1 accumulation on rice spikelets inoculated with Δ*FpfetC* at 21 dpi. (**D**) Relative expression levels of genes (*FpsidA*, *FpsidC*, and *FpsidF*) associated with siderophore biosynthesis at sites of inoculation challenged by Δ*FpfetC* at 48 h post-infection (hpi). Expression value of each gene in Fp9 strain was artificially set as 1. (**E**) Invasive hyphae on endepidermis of rice glumes infected with Δ*FpfetC* at 24, 48, and 72 hpi under scanning electron micrograph (SEM). Arrows indicate representative hyphae. Scale bars, 100 μm. (**F**) Ultrastructure of rice glumes challenged by Δ*FpfetC* at 72 hpi under transmission electron micrograph (TEM). Sg indicates starch grain. Chl indicates chloroplast. Scale bars, 500 nm. (**G**) Penetration of Δ*FpfetC* on cellophane membranes. Δ*FpfetC* was grown on MM media overlaid with cellophane membranes containing different concentrations of iron at 28 °C for 3 days (Before). After removing the cellophane, the plates were cultured for additional 3 days (After). Data are presented as mean ± standard deviation. Error bars denote standard deviation from three biological replicates. Asterisks indicate statistical significance as determined by using ANOVA followed by Student’s *t*-tests (ns—not significant; ***—*p* < 0.001). Each experiment was performed in triplicate.

Δ*FpfetC* mutant showed more severe necrotic lesions than the Fp9 strain after infecting rice spikelets (Figure 7A,B). FB1 accumulation on spikelets caused by Δ*FpfetC* was dramatically higher than the Fp9 strain (Figure 7C). Moreover, *FpsidA*, *FpsidC*, and *FpsidF* genes, responsible for synthesis of siderophore, were induced at the sites of inoculation challenged by Δ*FpfetC* (Figure 7D). The pathogenicity of Δ*FpfetC*-C had no difference to the Fp9 strain. To determine the effect of iron supply on virulence, conidial suspensions of the strains containing different concentrations of iron were injected into rice spikelets. No significant changes in plant phenotypes were detected among any of these strains with different levels of iron in conidial suspensions.

Compared to the Fp9 strain, invasive hyphae of Δ*FpfetC* were visible on endepidermis of glumes at 24 h post-infection (hpi) and formed dense hyphal structures on the epidermal tissues at 72 hpi (Figure 7E). The ultrastructure of infected glumes challenged by Δ*FpfetC* was abnormal, accompanying atypical accumulation of starch grains in the chloroplasts (Figure 7F). Under iron starvation (in the presence of BPS), the penetrating hyphae of Δ*FpfetC* were smaller than those of the Fp9 strain, but their phenotypes were similar when grown on no-iron (0 mM FeSO_4_) and low-iron (0.03 mM FeSO_4_) conditions (Figure 7G). As a whole, these results indicated that *FpfetC* had an important role in colonization and penetration during the initiation of infection.

## 3. Discussion

### 3.1. FpfetC Regulated Vegetative Growth in an Iron-Dependent Pattern in F. proliferatum

Filamentous fungi have evolved multiple modes to sequester or chelate trace amounts of iron, such as RIA system, SIA system, low-affinity iron uptake, and heme uptake pathways [23]. In the present study, as a core component of the RIA system, *FpfetC* was induced by iron starvation in *F. proliferatum*. Deletion of *FpfetC* led to impaired growth under iron deficiency, which was similar to poor growth at low levels of iron in *Candida parapsilosis* due to loss of *Fet3* [24]. In *Epichloë festucae*, disruption of *fetC* caused a slight decrease in colony growth under iron-deprived conditions, but the mutants lacking *sidA* (Δ*sidA*) or both *fetC* and *sidA* (Δ*fetC*/Δ*sidA*) could not grow normally, and exogenous iron fully restored colony phenotype of Δ*sidA* and partially rescued the defect of Δ*fetC*/Δ*sidA*, which was attributed to the existence of low-affinity iron uptake [25,26]. Low-affinity iron uptake system was also found in the rice false smut pathogen *Ustilaginoidea virens* [27]. Evidently, supplement of iron rescued the phenotype of Δ*FpfetC*. It was reasonable to surmise that reductive iron uptake was required for iron-dependent growth in *F. proliferatum*. Nevertheless, alternative iron acquisition pathways or nutrient recycling of iron, were able to engage for the compensatory effects for loss of RIA function.

### 3.2. FpfetC Was Essential for Transcription Regulation of Iron Uptake in F. proliferatum

Iron metabolism is an integrated architecture, which relies on intricate processes to modulate assimilation, utilization, storage, and excretion of iron [4]. With regard to *F. proliferatum* Fp9 strain, genes involved in iron acquisitions were induced under iron starvation, whereas genes associated with iron consumption and iron storage were repressed. The adaptive responses to iron deficiency emphasized that *F. proliferatum* adopted ferric/ferrous iron absorption and siderophore assimilation to acquire iron. The phenomenon was in agreement with the mode of iron sequestration in other fungi, such as *A. fumigatus* [28], *Fusarium graminearum* [29], *Colletotrichum heterostrophus* [30], *Nomuraea rileyi* [31]. These results indicated that the flexibility of iron uptake was necessary for alterations of iron homeostasis in the microenvironment.

**Figure 8:**
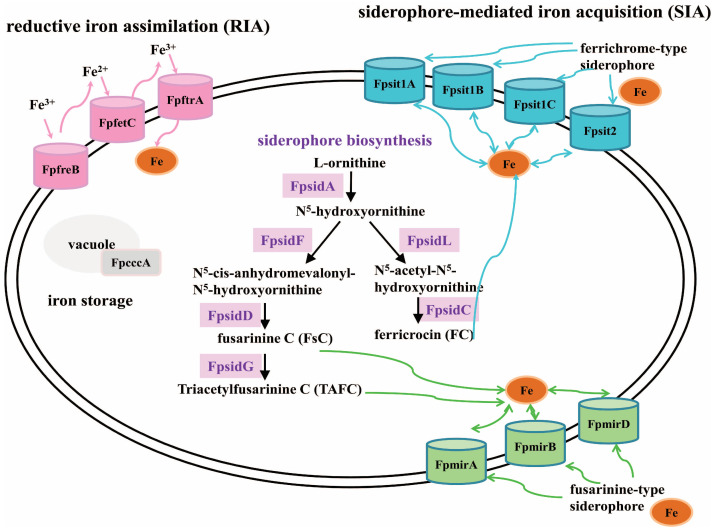
Schematic model for acquisition and transport of iron affected by *FpfetC* in *F. proliferatum*. Reductive iron assimilation is depicted in orange, composed of ferric reductase *(FpfreB*), multicopper ferroxidase (*FpfetC*), and iron permease (*FpftrA*). Siderophore biosynthesis enzymes are shown in purple, composed of ornithine-N^5^-oxygenase (*FpsidA*), non-ribosomal peptide synthetases (*FpsidC* and *FpsidD*), N^5^-transacylase (*FpsidF*), and transacetylases (*FpsidG* and *FpsidL*). Ferrichrome-type siderophore transporters are demonstrated in blue, composed of *Fpsit1A*, *Fpsit1B*, *Fpsit1C*, and *Fpsit2*. Fusarinine-type siderophore transporters are indicated in green, composed of enterobactin transporter (*FpmirA*), TAFC importer (*FpmirB*), and fusarinine C transporter (*FpmirD*). Vacuolar iron storage protein *FpcccA* is represented in gray. Environmental (chelated) iron is shown in brown dots.

Whether loss of *FpfetC* impacted the expression of iron-responsive elements was also tested. Remarkably, *F. proliferatum* lacking *FpfetC* resulted in perturbations of iron metabolism under both iron-depleted and iron-replete conditions. In the RIA system, the expression of *FpftrA* gene was obviously increased in Δ*FpfetC* mutant, which was congruent with *S. cerevisiae*, the absence of Ftr1 protein led to accumulation of Fet3 protein [32]. Siderophores are low-molecular weight molecules with high affinity for ferric iron [4]. Of these, fusarinine C (FsC) and its acetylated derivative triacetylfusarinine C (TAFC) are employed for extracellular iron handling, while ferricrocin (FC) and hydroxyl ferricrocin (HFC) are required for intracellular iron storage and distribution [3]. After the chelation of iron, the ferric–siderophore complexes are recognized and transported by the membrane transporters [4]. As for siderophore biosynthesis*,* expression of *FpsidA* gene encoding the first enzyme in the biosynthesis of siderophore and *FpsidD* gene responsible for the biosynthesis of FsC and TAFC, were drastically downregulated in Δ*FpfetC* mutant. In terms of siderophore transport, transcripts of FC transporters (*FpsitA*, *Fpsit1B* and *Fpsit1C*) and TAFC transporter (*FpmirB*) were decreased, whereas transcripts of enterobactin transporter (*FpmirA*) and FsC transporter (*FpmirD*) were increased in Δ*FpfetC* mutant. The data indicated that *FpfetC* was involved in biosynthesis and uptake of extra- and intracellular siderophores. Iron homeostasis is maintained by iron-sensing transcription factors HapX and SreA, which are interconnected in a negative feedback loop [4]. As expected, *FphapX* was induced under iron starvation, but was repressed under iron sufficiency in Fp9 strain, whereas *FpsreA* showed the opposite behavior. Lack of *FpfetC* led to downregulation of *FphapX* in iron starvation and *FpsreA* in iron sufficiency. As a means of iron detoxification, iron is transported into the vacuole for storage by vacuolar iron importer Ccc1 [33]. Under iron-replete conditions, expression of *FpcccA* (ortholog of *ccc1*) was upregulated in Fp9 strain, whereas it was downregulated in Δ*FpfetC*. Overall, these findings reinforced that *FpfetC* played a crucial role on transcription regulation of genes involved in high-affinity iron uptake, iron homeostasis, and vacuolar-mediated iron storage. According to the overview of transcription remodeling, an upgraded framework of regulatory rewiring of iron acquisition and transport mediated by *FpfetC* was postulated in *F. proliferatum* (Figure 8).

### 3.3. FpfetC Was Involved in Response to Copper and Zinc Stresses in F. proliferatum

As the redox-active metals, copper and iron are often tightly interwoven in metabolism. In *S. cerevisiae*, copper was obligatory for the RIA system, multicopper oxidase Fet3 depended on the donation of copper by P-type ATPase Ccc2, because copper ion was delivered from copper transporter Ctr1p to copper transport ATPase Ccc2 [34]. *Ccc2*-deficiency almost abolished iron uptake due to inactivation of copper-dependent oxidase that was required for extracytosolic domain of the ferroxidase [35]. Under high levels of copper, *crpA* gene (homolog of *Ccc2*) of *Aspergillus nidulans* was induced by copper-binding transcription factor AceA, which was needed to pump copper out of the cytoplasm to avoid the toxicity of copper [36]. The *crpA* and *crpB* genes were involved in tolerance to copper in *Aspergillus flavus*; deletion of both genes led to sensitivity to copper [37]. Here, absence of *FpfetC* increased resistance to copper stress; concomitantly, expression of *Fpccc2* gene (ortholog of *ccc2*) was significantly upregulated. The results demonstrated that *F. proliferatum* might evolve coordination between iron and copper uptake to guarantee metabolism reprogramming.

Loss of *FpfetC* rendered *F. proliferatum* more sensitive to excess zinc, with the decline in transcript levels of zinc transporters (*Fpzrts*) after exposure to a high concentration of zinc. Previous studies demonstrated that zinc acquisition was fine-tuned in cooperation with iron uptake. In *S. cerevisiae*, low-affinity iron transporter *FET4* was induced by transcription factor Zap1 involved in zinc homeostasis under zinc-limiting conditions [38]. Also, for *Paracoccidioides* spp., though it did not present high-affinity iron permease (Ftr1), the fungus relied on zinc transporters (zrts) to import zinc and iron into the cytoplasm under iron-deprived conditions [39]. In *A. fumigatus*, iron deficiency led to fluctuations of intracellular zinc influx by orchestrating upregulation of vacuolar zinc transporter and downregulation of zinc importer [40]. Transcription factor ZafA (homolog of Zap1) not only regulated zinc transport but also participated in iron uptake under zinc starvation [41]. The growth of *A. fumigatus* was more obviously affected by the levels of iron in zinc-replete than that in zinc-limiting conditions, which depended on transcription regulation of ZafA [42]. As such, we speculated that there was a crosstalk between iron and zinc metabolism in *F. proliferatum* and it was proposed; however, the interdependent impacts of metal ions remain to be elucidated.

### 3.4. FpfetC Negatively Regulated Fumonisin FB1 Production in F. proliferatum

Fungi can produce a wide variety of secondary metabolites in occupying favorable ecological niches [43]. Fungal secondary metabolism is a sophisticated and hierarchical regime, which is operated by global and pathway-specific regulators, epigenetic control, and chemical or environmental stimuli [44]. Iron uptake and transport have been regarded as environmental signals associated with secondary metabolism. In *A. fumigatus*, deletion of *PpzA* gene encoding a catalytic subunit of phosphatase Z, that was involved in iron acquisition, led to reductions in pyripyropene A, fumagillin, fumiquinazoline A, triacetyl-fusarinine C, and helvolic acid [45]. In the entomopathogenic fungus *Metarhizium robertsii*, inactivation of Sfp-type 4′ phosphopantetheinyl transferase (*mrpptA*), responsible for acquisition of ferricrocin, failed to produce the nonribosomal peptides and hybrid polyketide-peptides [46]. In *Candida albicans*, Rim101-upregulated ferroxidases (Fets) contributed to formation of dark-pigmented melanin [47]. In this context, loss of *FpfetC* provoked FB1 production in *F. proliferatum. FpfetC*-deficiency enhanced the expression of key genes responsible for fumonisin biosynthesis, including *Fpfum21* gene participating in the cluster-specific regulation, *Fpfum1* gene responsible for assembling the 18-carbon polyketide chain, *Fpfum6* gene hydroxylating the C-14 and C-15 positions of polyketide-amino acid condensation, and *Fpfum8* gene catalyzing the condensation reaction between the alanine and polyketide to generate 20-carbon fumonisins. These findings corroborated that *FpfetC* was a negative modulator of fumonisin cluster-specific biosynthesis in *F. proliferatum*.

### 3.5. FpfetC Was Crucial for the Pathogenicity in F. proliferatum

Pathogens are able to use different patterns of iron uptake for successful infection in hosts [48]. Targetting iron utilization might be an effective option for antifungal chemotherapy, since both iron deprivation and iron overload was detrimental to fungi [49,50]. Application of the synergistic effect of iron chelators and antifungal drugs was shown to be a potential avenue for treatment of *A. fumigatus* [51]. Inactivation of *FpfetC* resulted in severely rotten symptoms on rice spikelets, in conjunction with propagation of invasive hyphae inside epidermal cells of rice glumes. Hyphae formation of *F. proliferatum* played a critical role in the initial colonization of rice [52]. The tiny conidia were able to germinate from the differentiated hyphae as the primary means of reproduction. *FpfetC*-deficiency produced a massive number of conidia during asexual development. Upon entry into the host, these attributes were corollaries to colonization and transmission of the pathogen. Our data were consistent with the findings of ferroxidase in gray mold pathogen *Botrytis cinerea*; abrogation of *bcfet1* caused high virulence [53]. By contrast, in the opportunistic human-pathogenic fungus *C. albicans*, deletion of ferroxidase attenuated virulence in murine model of oropharyngeal candidiasis [54]. In the hemibiotrophic pathogen *C. graminicola*, the mutant of ferroxidase failed in biotrophic growth on maize [6]. The most likely interpretation for this discrepancy was that ferroxidase performed distinct roles on pathogenicity in different microbial systems.

After establishing the compatible interaction, the toxigenic fungi were able to produce mycotoxins to interfere with host immune responses [55]. Certain mycotoxins, such as aflatoxins, ochratoxin, deoxynivalenol (DON), trichothecenes, and *Alternaria* host-specific toxins, resulted in the deleterious effects on the hosts [56,57,58]. In *F. graminearum*, the DON-producing strains enhanced their ability to invade the wheat more effectively than the non-DON-producing strains [59]. DON induced lipid peroxidation, leading to inhibition of DNA synthesis and cell membrane dysfunction [60]. Fumonisin FB1 as a virulence determinant has been characterized in *Arabidopsis thaliana*, whereby FB1 caused cell death through generation of hydrogen peroxide, callose deposition, and damage to membrane lipids [61]. *F. proliferatum* was capable of producing fumonisins to weaken or kill different hosts for the transition from the hemibiotrophic to saprophytic stage [14,62,63]. It was previously demonstrated that fumonisins were required for the exacerbation of virulence of *F. proliferatum* to rice [64], banana fruit [65], and maize, sorghum, and pearl millet [66]. Intriguingly, FB1 accumulation on rice spikelets was remarkably elevated after inoculation of Δ*FpfetC* mutant. One possibility was that this strong pathogenicity was due, at least partially, to mycotoxin production in the interaction with rice.

Siderophore biosynthesis and transport played crucial roles in virulence of fungal pathogens, as shown in *E. festucae* [26], *N. rileyi* [31], *A. fumigatus* [67], and *Alternaria alternata* [68]. In *C. graminicola*, siderophore synthesis acted as a decision-making event in necrotrophic phase [69]. Activation of siderophore synthesis at infected tissues was challenged by Δ*FpfetC*, which indicated that intracellular siderophore-assisted iron mobilization accounted for the main route for iron availability. The investigation in *M. robertsii* also supported the idea that iron sequestration through intracellular siderophores was required for full virulence [70]. In contrast to *Cochliobolus heterostrophus*, the defect of RIA components displayed virulence as wild type, but loss of extracellular, not intracellular, siderophores did attenuate virulence [30]. These results implied that the synthesis and transport of siderophores employed by *F. proliferatum* was involved in the infection processes.

Taken together, hypervirulence caused by Δ*FpfetC* was the consequence of multiple factors, including aggravation of invasive hyphae, accumulation of fumonisins production, and utilization of siderophore biosynthesis. There is the possibility for the development of metal-based fungicides or therapeutic interventions, which contributes to the prevention or control of *F. proliferatum*-induced diseases in crops.

## 4. Materials and Methods

### 4.1. Fungal Strains and Culture Conditions

The *F. proliferatum* strain Fp9 was originally isolated from a naturally infected sample of rice spikelet rot in China [14]. The Fp9 strain was used as wild type (WT) for generation of the genetically modified strains. The fungal strains were stored as conidial suspensions in 30% glycerol stocks at −80 °C. For the growth assay, the strains were cultivated on PDA media (200 g of potato, 20 g of glucose, and 15 g of agar per liter) [71], CM media (10 g of glucose, 2 g of peptone, 1 g of yeast extract, 1 g of casamino acids, 0.5 g of KCl, 6 g of NaNO_3_, 1.5 g of KH_2_PO_4_, 1 g of MgSO_4_·7H_2_O, 1 mL of iron-free trace elements, and 20 g of agar per liter, pH 6.5), and MM media (30 g of sucrose, 20 mM of glutamine, 1 g of KH_2_PO_4_, 0.5 g of MgSO_4_·7H_2_O, 0.5 g of KCl, 200 μL of trace elements, and 20 g of agar per liter, pH 6.9). The trace elements solution was consisted of 50 g of ZnSO_4_·7H_2_O, 50 g of citric acid, 2.5 g of CuSO_4_·5H_2_O, 0.5 g of MnSO_4_·H_2_O, 0.5 g of H_3_BO_3_, and 0.5 g of Na_2_MoO_4_·2H_2_O per liter. For conidiation assay, the strains were incubated in YEPD media (10 g of yeast extract, 20 g of peptone, and 20 g of glucose per liter) [72]. Conidia were collected through sterile cheesecloth to remove mycelia and quantified by a hemocytometer. To test the response to iron, the strains were grown on MM media omitting iron, then transferred to MM media supplemented with iron chelator BPS (Sigma-Aldrich, St. Louis, MO, USA) at a final concentration of 0.3 mM or FeSO_4_·7H_2_O at final concentrations of 0.03 mM, 1 mM, 5 mM, or 10 mM, respectively. To assess the sensitivity to metal ions, the strains were inoculated on PDA plates supplemented with final concentrations of 0.2 mM CuSO_4_ or 10 mM ZnSO_4_.

### 4.2. Phylogenetic Analysis

The amino acid sequence of Fet3 (GenBank accession no. NP_013774.1) of *S. cerevisiae* was used as the query to search for orthologous protein sequence in the dataset of *F. proliferatum* ET1 strain (GenBank accession no. FJOF00000000) using the NCBI BLASTP algorithm. A conserved domain feature was detected through Conserved Domain Database (CDD) [73]. The amino acid sequences of homologous proteins from other fungal species were retrieved from the NCBI database. The sequences were aligned with Clustal X program [74]. A phylogenetic tree was constructed using the neighbor-joining method with 1000 bootstrap replicates using MEGA11 software (version 11.0) [75].

### 4.3. Gene Deletion and Complementation

Gene deletion was conducted using the homologous recombination strategy [76]. Upstream (5′-) and downstream (3′-) flanking regions were amplified from the Fp9 strain with primers F1/R1 and F2/R2, respectively. The hygromycin B phosphotransferase gene (*HYG*) was amplified with primers F3/R3 using plasmid pFGL821 as a template. Partial *HYG* designated as HY and YG were fused with 5′- and 3′-flanking regions with primers F4/R4 and F5/R5, respectively. Overlapping but incomplete fragments were transformed into Fp9 strain using polyethylene glycol (PEG)-mediated transformation [64]. Transformants were screened on PDA medium with 200 μg/mL hygromycin B (Calbiochem, San Diego, CA, USA) and identified by diagnostic PCR using locus-specific primers F6/R6 and F7/R7 and *FpfetC*-specific primers F8/R8. Purified transformants were confirmed by Southern blotting analysis. The hybridization probe was amplified with primers F9/R9. The procedures for probe labeling, hybridization, and detection were performed according to DIG DNA Labeling and Detection Kit (Roche, Mannheim, Germany).

To generate complementary strain, a full-length *FpfetC* gene fused to geneticin (G418)-resistance gene (*GEN*) was reintroduced into Δ*FpfetC*. Briefly, *FpfetC* gene with the native promoter and terminator connected with 5′-flanking region was amplified with primers Com-F1/Com-R1. The 3′-flanking region was amplified with primers Com-F2/Com-R2. The *GEN* gene was amplified from plasmid pN580 with primers Com-F3/Com-R3. Partial *GEN* genes designated as GE and EN fused with 5′- and 3′-flanking regions were amplified with primers Com-F4/Com-R4 and Com-F5/Com-R5, respectively. Two fragments containing overlapping sequences were transformed into ∆*FpfetC*. Transformants resistant to G418 (LifeTechnologies, Carlsbad, CA, USA) were detected by diagnostic PCR. Single-copy integration was validated by qRT-PCR [77]. All primers used for the construction and verification of the mutants are listed in Table S1. Schematic representations of gene deletion and complementation are illustrated in Figure S1 and Figure S2, respectively.

### 4.4. RNA Isolation and Quantitative Real-Time PCR (qRT-PCR)

Total RNA was extracted using Trizol Reagent (Invitrogen, Carlsbad, CA, USA). The concentration and purity of RNA was determined with a NanoDrop ND-1000 spectrophotometer (Thermo Fisher Scientific, Waltham, MA, USA). RNA was reverse transcribed into cDNA using PrimeScript^TM^ RT reagent Kit with gDNA Eraser (Takara, Otsu, Shiga, Japan). qRT-PCR was performed by SYBR Premix Ex Taq^TM^ (Takara, Otsu, Shiga, Japan) with a QuantStudio 3 Real-Time PCR system (Applied Biosystems, Carlsbad, CA, USA). Relative expression value was calculated with the delta-delta comparative threshold (2^−ΔΔCT^) method [78]. The *β-tubulin* (*Fptub*) gene of *F. proliferatum* was used as internal control for normalization. All primers used for qRT-PCR amplification are listed in Table S2.

### 4.5. Determination of FB1 Production

The strains were inoculated in PDB media or cracked rice kernels. FB1 content was measured by high performance liquid chromatography–tandem mass spectrometry (HPLC-MS/MS) [13]. Briefly, samples were extracted using an acetonitrile/water (1:1, *v*/*v*) solvent overnight at room temperature. The mixture was filtered through a nylon syringe filter. The chromatographic separation was performed with a HPLC system (Thermo Fisher Scientific, Waltham, MA, USA) equipped with a Zorbax Extend-C18 column (100 mm × 2.1 mm, 3.5 μm). The mobile phase consisted of methanol/water/formic acid (75:25:0.2, *v*/*v*/*v*) at a flow rate of 300 μL/min. FB1 was detected with mass spectrometer (MS) equipped with electrospray ionization (ESI) in positive ion mode. The following MS settings were applied: source temperature of 120 °C, desolvation temperature of 350 °C, flow rate of 600 L/h, capillary voltage of 3500 V. Robustness of HPLC-MS/MS was evaluated by changing the procedural conditions. Performance characteristics were validated as described previously [79,80]. The evaluated parameters included linearity, matrix effect, limit of detection (LOD), limit of quantification (LOQ), recovery, intra-day precision (repeatability), and inter-day precision (reproducibility). To verify linearity, the calibration curve was established by FB1 standard (Sigma-Aldrich, Saint Louis, MO, USA) at seven concentration levels from 5 μL/kg to 500 μL/kg. The linearity was evaluated based on correlation coefficient (*r*^2^). Matrix-matched calibration curves were built by spiking blank samples with FB1 samples after the extraction process. Matrix effect was obtained by comparing the slope of matrix-matched calibration curve with that of standard calibration curve. Sensitivity was evaluated by determining LOD and LOQ as a signal-to-noise ratio (S/N) of 3 and 10, respectively. The recovery was assessed by the ratio of the peak areas for the samples spiked before and after extraction at three concentrations (low, median and high). The precision was demonstrated as repeatability and reproducibility with the fortified samples spiked at medium concentration level.

### 4.6. Plant Infection Assay

A pathogenicity test was carried out with rice susceptible cultivar Jiahe218, as described previously [81]. Conidia of the strains were collected from 4-day-old cultures grown in YEPD media and adjusted to a final concentration of 10^6^ conidia/mL. To test the fungal virulence, a conidial suspension was injected into a spikelet until overflow was reached at the booting stage [82]. The inoculated plants were placed in the greenhouse at 28 °C with relative humidity of 85% under photoperiod of 14 h-light/10 h-dark. At 21 days after inoculation, disease severity was investigated and disease index was calculated [81]. Ten plants were inoculated for each treatment, and the experiment was conducted three times independently. To evaluate the fungal ability to colonize rice, a 10-μL aliquot of conidial suspension was injected into a floret at the anthesis stage [13]. The inoculated plants were maintained with relative humidity of 85% at 28 °C in the growth chamber. Microscopic observations of inoculated tissues were conducted with scanning electron micrograph (SEM) and transmission electron micrograph (TEM) [83,84]. To detect penetration ability of hyphae, the strains were inoculated on a cellophane membrane laid on MM plates supplemented with BPS or FeSO_4_·7H_2_O at 28 °C for 3 days. After removing the cellophane with the fungal colony, plates were cultured for an additional 3 days to examine whether hyphae appeared on the media.

### 4.7. Scanning Electron Microscopy (SEM)

The samples were fixed with 2.5% glutaraldehyde in 0.1 M phosphate buffer (PBS, pH 7.0) at 4 °C for 24 h, washed three times with PBS, postfixed with 1% OsO_4_ for 2 h, and dehydrated in a graded series of ethanol (30%, 50%, 70%, 80%, 95%, and 100%) for 15 min at each step. The samples were transferred to pure isoamyl acetate overnight, and critical-point dried in a Hitachi Model HCP-2 critical point dryer with liquid CO_2_ (Hitachi High-Technologies Corporation, Tokyo, Japan). Subsequently, the samples were sputter-coated with gold using a Hitachi Model E-1010 ion sputter (Hitachi High-Technologies Corporation, Tokyo, Japan). The fungal hyphae were observed under a Hitachi Model SU-8010 SEM (Hitachi High-Technologies Corporation, Tokyo, Japan).

### 4.8. Transmission Electron Microscopy (TEM)

The samples were fixed with 2.5% glutaraldehyde in 0.1 M PBS at 4 °C for 24 h. After washing with PBS, the samples were postfixed with 1% OsO_4_ for 2 h and serially dehydrated by immersion in ethanol solutions of 30%, 50%, 60%, 70%, 80%, 90%, and 100% for 15 min each time. Then, samples were placed in a mixture of acetone and Spurr resin (1: 1, *v*/*v*) for 1 h, transferred into a mixture of acetone and resin (1: 3, *v*/*v*) for 3 h, and placed in final Spurr resin for overnight. After polymerization at 70 °C for 9 h, the specimens were sectioned by a Leica EM UC7 ultramicrotome (Leica Microsystems, Buffalo Grove, IL, USA). Ultrathin sections were double-stained with uranyl acetate and alkaline lead citrate for 10 min, respectively. Ultrastructure of rice glumes were observed under a Hitachi Model H-7650 TEM (Hitachi High-Technologies Corporation, Tokyo, Japan).

### 4.9. Statistical Analysis

Statistical analysis was performed using GraphPad Prism version 8.4.3 for Windows (GraphPad software, La Jolla, CA, USA). For all experiments, data were presented as the means and standard deviation with three biological replicates. Difference among treatments was determined using one-way analysis of variance (ANOVA) followed by Student’s *t*-test. Significance was denoted by *p*-values (ns—not significant; *—*p* < 0.05; **—*p* < 0.01; ***—*p* < 0.001). Different lowercase letters indicated the statistically significant differences as determined by ANOVA with Student’s *t*-tests at *p* < 0.05.

## 5. Conclusions

In summary, this study demonstrated the roles of multicopper ferroxidase in *F. proliferatum*. The pleiotropic functions of *FpfetC* were not limited to iron assimilation, but were also related to conidiation, mental stress tolerance, FB1 biosynthesis, and pathogenicity. Considering the potentiality of *FpfetC* as a regulator for virulence and fumonisins, *FpfetC* might be an attractive target for antifungal drugs, which is beneficial for development of management strategies against *F. proliferatum*.

## Data Availability

The original contributions presented in the study are included in the article/Supplementary Material; further inquiries can be directed to the corresponding authors.

## Supplementary Materials

The following supporting information can be downloaded at: https://www.mdpi.com/article/10.3390/ijms26072883/s1.
