# Supplementary material for: The Role of FpfetC from Fusarium proliferatum in Iron Acquisition, Fumonisin B1 Production, and Virulence
Source: Int J Mol Sci. 2025 Mar 22;26(7):2883. doi: 10.3390/ijms26072883 (PMC11988320; doi:10.3390/ijms26072883)
Supplement: Supplementary file 1 [file ijms-26-02883-s001.zip › ijms-3448722-supplementary.pdf]

Supplementary Materials

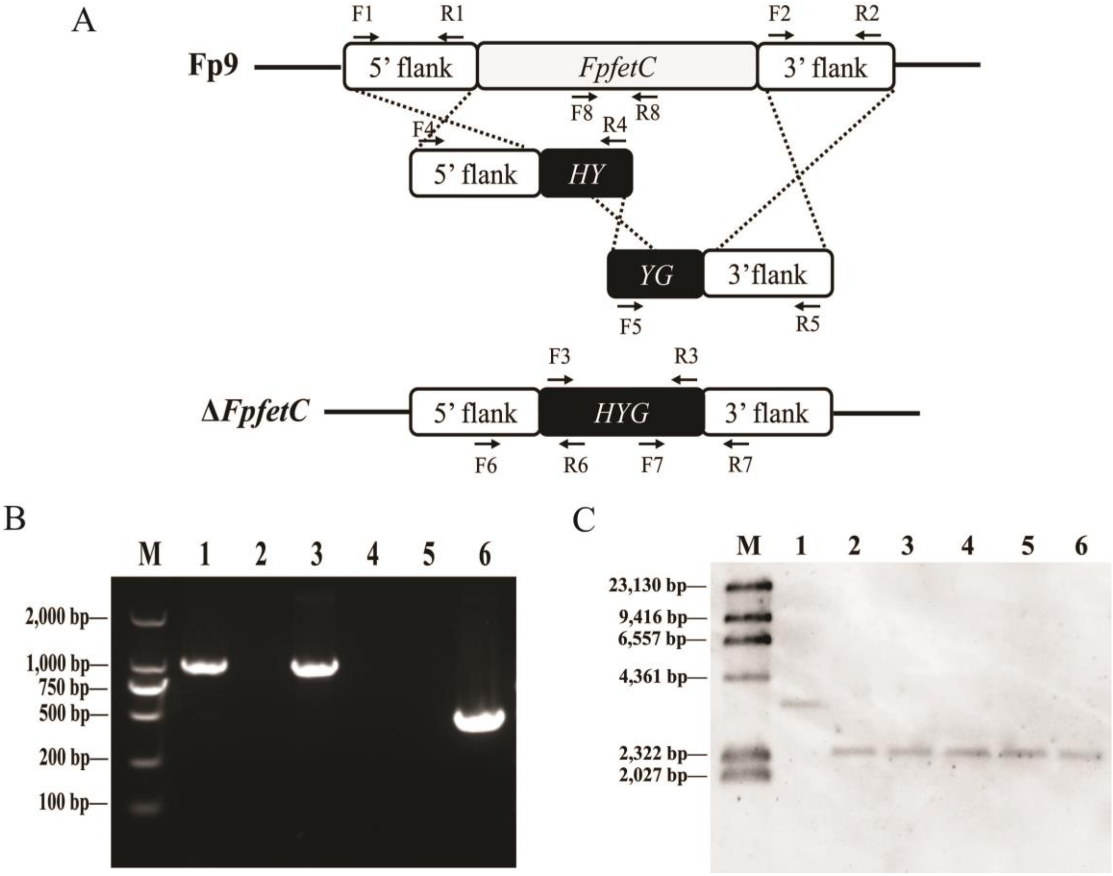

Figure S1. Deletion of *FpfetC* gene in *F. proliferatum*. (**A**) Diagram of deletion of *FpfetC* gene using homologous recombination. Hygromycin resistance gene (*HYG*) was used to replace *FpfetC* gene to generate Δ*FpfetC* mutants. *HY* and *YG* showed partial regions of *HYG* gene, respectively. Black arrows indicated the position and orientation of the primers. (**B**) Electrophoresis gel of PCR products confirming deletion of *FpfetC* gene in Fp9 strain. Homologous integrations at 5′- and 3′- flanking regions were detected in Δ*FpfetC* (lanes 1 and 3) but not in Fp9 strain (lanes 2 and 4) with primers F6/R6 and F7/R7, respectively. *FpfetC* gene was detected in Fp9 strain (lane 6) but not in Δ*FpfetC* (lane 5) with primers F8/R8. M, DL 2,000 DNA marker. (**C**) Southern blot analysis of Δ*FpfetC* mutants. Genomic DNA was digested with *BamH*I and hybridized with DIG-labeled probe. The upstream fragment of *FpfetC* gene was used as the probe. Expected size of 3,341 bp was detected in Fp9 strain (lane 1), while anticipated size of 2,351 bp was detected in the five independent mutants of Δ*FpfetC* (lanes 2 to 6).

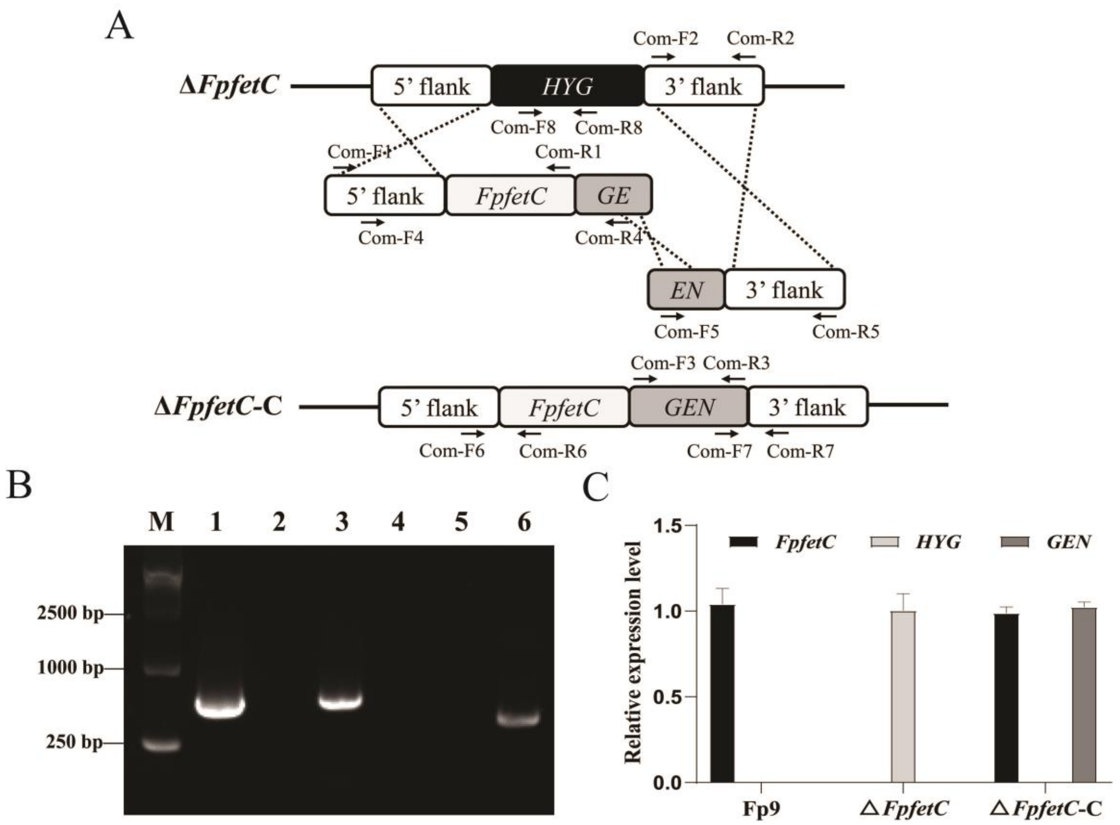

Figure S2. Generation of complementary strain Δ*FpfetC*. (**A**) Diagram of *FpfetC* gene and geneticin-resistance gene (*GEN*) reintroduced into Δ*FpfetC* mutants. *GE* and *EN* showed partial regions of *GEN* gene. Black arrows indicated the position and orientation of the primers. (**B**) Electrophoresis gel of PCR products confirming generation of Δ*FpfetC*-C. Homologous integrations at the 5′-region and 3′-region were detected in Δ*FpfetC*-C (lanes 1 and 3) but not in Δ*FpfetC* (lanes 2 and 4) with primers Com-F6/Com-R6 and Com-F7/Com-R7, respectively. *HYG* gene was detected in Δ*FpfetC* (lane 6) but not in Δ*FpfetC*-C (lane 5) with primers Com-F8/Com-R8. M, DL 15,000 DNA marker. (**C**) Relative expression level of three genes (*FpfetC*, *HYG*, *GEN*) in Fp9, Δ *FpfetC* and Δ *FpfetC*-C. Expression value of *FpfetC* gene in Fp9 strain was artificially set as 1. Error bars denoted standard deviation from three biological replicates.

Table S1. Primers used for gene deletion and complementation in this study

Primer name	Primer sequence (5′-3′)	Application	Purpose
F1	GGGTGATGACCTGGTGGAGAAATAACATTATACTATCCGTATAT	amplify *FpfetC* 5′ flank sequence	
R1	GCTTTTTCATCGCGGCAGTCACTAGTTCA	amplify *FpfetC* 5′ flank sequence	
F2	AAAGAAATAGAGAGAATATTATACGTGTATCATAT	amplify *FpfetC* 3′ flank sequence	
R2	GGCCTGCGCGGCCTTCAACTTACTAATGACGATGAATAGCCA	amplify *FpfetC* 3′ flank sequence	
F3	GACTGCCGCGATGAAAAAGCCTGAACTCACCGC	amplify *HYG* sequence	Generation of deletion mutant
R3	AATATTCTCTCTATTTCTTTGCCCTCGGACG	amplify *HYG* sequence	
F4	TAAAGATACCCCAGCCACCGCCACC	amplify 5′ flank and *HYG* gene	
R4	CTGCTGCTCCATACAAGCCAACC	amplify 5′ flank and *HYG* gene	
F5	ATCGTTATGTTTATCGGCACTTTG	amplify 3′ flank and *HYG* gene	
R5	AGAGGAGATCAACCATCACCACCAA	amplify 3′ flank and *HYG* gene	
F6	AAGTCATCTGCCTGTAAA	Detection of left boarder of deletion mutants	
R6	GACCGTCTGGGTATTGTG	Detection of left boarder of deletion mutants	
F7	GGTAGGTGTCTGGCTCTT	Detection of right boarder of deletion mutants	
R7	TGTAATGTTTCGACGGTG	Detection of right boarder of deletion mutants	Confirmation of deletion mutants
F8	TCTGGATCGAAGGGCATA	Detection of *FpfetC* gene of deletion mutants	
R8	GAAGTTTTGAAGGGCTGT	Detection of *FpfetC*gene of deletion mutants	
F9	TCAAAGTCATCTGCCTGT	Construction of DIG-labeled probe for Southern blot	
R9	CCACACACTACCGAAATC	Construction of DIG-labeled probe for Southern blot	

Table S1. Primers used for gene deletion and complementation in this study (continued)

Primer name	Primer sequence (5′-3′)	Application	Purpose
Com-F1	TTCCGATCCCCAGGGCAGTGCCTGTAAATGATGTATCCAACAACG	amplify 5′ flank and *FpfetC* gene	Generation of complementary strains
Com-R1	GTTCTTCTGATTACTTGGCTGCAATGGGTG	amplify 5′ flank and *FpfetC* gene	
Com-F2	CAATCCCCATAGAGAATATTATACGTGTATCATAT	amplify 3′ flank sequence	
Com-R2	CACGGCCGCCCAATCGCGGGGTTCATAAATGGATAATGGACAGT	amplify 3′ flank sequence	
Com-F3	AGCCAAGTAATCAGAAGAACTCGTCAAGAAGGC	amplify *GEN* sequence	
Com-R3	AATATTCTCTATGGGGATTGAACAAGATGGATTGC	amplify *GEN* sequence	
Com-F4	ATTGCCGCAGTCTTTATC	amplify 5′ flank, *FpfetC* and *GEN* gene	
Com-R4	TCTCCTGTCATCTCACCT	amplify 5′ flank, *FpfetC* and *GEN* gene	
Com-F5	GTAAAGCACGAGGAAGCG	amplify 3′ flank and *GEN* gene	
Com-R5	CAGAAGAAGGTCAAGGCA	amplify 3′ flank and *GEN* gene	
Com-F6	GCTGGGATTTCGGTAGTG	Detection of left boarder of complementary strains	Confirmation of complementary strains
Com-R6	TCTTGGATGATGAGGGCT	Detection of left boarder of complementary strains	
Com-F7	GGCAGGAGCAAGGTGAGA	Detection of right boarder of complementary strains	
Com-R7	GGGTCCAGATGCGAGAGC	Detection of right boarder of complementary strains	
Com-F8	CATTGGGGAGTTTAGCGA	Detection of *HYG* gene of complementary strains	
Com-R8	CAGAAGAAGATGTTGGCG	Detection of *HYG* gene of complementary strains	

Table S2. Primers for qRT-PCR in this study

Gene name	Forward sequence (5'-3')	Reverse sequence (5'-3')	Annotation
FpfetC	CACAGACGGTGAGAAGT	GTTGAAGAAGGCATAGTT	ferroxidase
FpfreB	TAATCTCCTTATGTGGCTAA	ACCCGACAGAGTGAACG	ferric reductase
FpftrA	AAACGCCTGCTCAAAC	TGCCGAGGGAGTAGAA	iron permease
FpsidA	TAACTACCTCCACCGTCAG	AACCAAGAAGAGCACCAG	ornithine-N^5-oxygenase
FpsidC	CAGGAAACGGACATACC	ACTTCATCTGCACCAGTC	non-ribosomal peptide synthetase
FpsidD	GCGAAACCAGAATAAGGA	CAGACGAGGTGGGAAGT	non-ribosomal peptide synthetase
FpsidF	GGGAGAAGCGTCAAACC	TCTGGGCACCAAACAGG	N^5-transacylase
Fpsit1A	TCGGCGTCTTCCTAATCG	GGTATTGACGGAGGACTGGA	siderochrome-iron transporter
Fpsit1B	CGGGTTCTTCGCTTATCC	TAGGGCACTTCCGACATT	siderochrome-iron transporter
Fpsit1C	CGCTCGGGTACAGAATC	GGCCAGTTAGGCACAAA	siderochrome-iron transporter
Fpsit2	CTCCCAATGAGACCACCC	AACCTCCTGCTCGTTCCT	siderochrome-iron transporter
FpmirA	CGTGACGGGCGTTTACT	TTGGGCTTTGCTTGCTC	enterobactin transporter
FpmirB	AAGGGAACAGTCACCAAG	AGAGGAAGATGCCGATAA	TAFC transporter
FpmirD	GGTTTCCAGAGCCAGAT	TAGACAGCGGCAGTCAT	fusarinine C transporter
FpcccA	TGCACCTTTAGTAGACGA	ATGGTAACCGCTGAGAT	vacuolar iron importer
FphapX	CCCGCAAGACTCAAGAA	CGCAGACGCAGTAGGTT	bZIP transcription factor
FpsreA	CTCCCATACGATCACCC	CGCTCAGCCTTTATTCTC	GATA transcription factor
FpestB	CGGACACGAGTGAGATTAT	GGAGCCGATGTTGAAGT	triacetylfusarinine C esterase
FplysF	CGCAACAGCATCAACAA	CCCAGACAAACTTCCACC	homoaconitase
FphemA	AGCCTACTTCTGCTTCTCC	AGCGGTATGATTTGTCCTT	5-aminolevulinate synthase
FpcycA	ACGGCGGCAACAAGATC	TGTCGTCGTTCCACTCAAT	cytochrome C
FpacoA	CCCGTGATGCCCTCAAC	CGATACCGCCGAACTCC	aconitate hydratase
FpbrlA	TCGTGAGATGCCTGTCGT	TTCGCAAGTCGGAGAAGA	C2H2 transcription factor
FpabaA	TCAGGTGCCAAATGTTCC	GTCAAAGCTGGTCGGGTA	transcription factor of conidiophore development

Table S2. Primers for qRT-PCR in this study (continued)

Gene name	Forward sequence (5'-3')	Reverse sequence (5'-3')	Annotation
FpwetA	CACCCAGACCTACCTCCCT	CAAGACAAGTGTCGTGCTCC	transcription factor of conidiophore development
Fpccc2	CGGACCAAATAGCGGAAAT	GAACTGTGCCGTGGACGA	copper transport ATPase
Fpzrt1	CAGCAACCCTGACATCGA	CCTGAGCATCACCCTCCT	zinc regulated transporter
Fpzrt2	AACATGGTCAACGGTATTT	CCTTGTCATTCGTCCTCT	zinc regulated transporter
Fpzrt3	ATGATGCCCAGCAAACTC	GGTTACCGAACATGAGAAAT	zinc regulated transporter
Fpzrt4	GGCGGAGAAGTCCAAGTT	TCTGACACGGCAGTGATC	zinc regulated transporter
Fpfum1	CCAACTCTTCTTCCCTGCTA	CACCCTCTACCTCCCACA	polyketide synthase
Fpfum3	CCACGACCGATTTCACAG	TCCAGCCTTCATTTCAGC	dioxygenase
Fpfum6	CTGGAAAGTATGCGGTCAA	GCAGAACTCATCAGCGTCA	cytochrome P450 monooxygenase
Fpfum7	CAGGGCGGTTGACCACTT	GCGACGCCTGGATGTCTT	dehydrogenase
Fpfum8	GCGGAACGAGAAATAGTGA	TGCTGGGTTGAAAGGGAG	aminotransferase
Fpfum10	ACGGAATGACTGAGACGG	GGGAATCGGGTATTGACC	fatty acyl-CoA synthetase
Fpfum11	AAGGTGTTCTCGCCCTCT	ACCCTCCATCACTTTCTCAT	tricarboxylate transporter
Fpfum12	CGACGGCTACACTGCTTA	GCTCCTCACTAGACCCAAA	cytochrome P450 monooxygenase
Fpfum13	TCCGGGCAGCTCAGAATT	GGCGTGATACAGCGACCA	short-chain dehydrogenase/reductase
Fpfum14	TCAAGCTCGCCTCCTACCAC	CCACGATGACCGACTATCCC	peptide synthetase condensation domain
Fpfum15	AACCTCTACCCTATCTTCTGG	TGACTGTCTCCGTACCTGA	cytochrome P450 monooxygenase
Fpfum16	CCTTACAGATGCGTCCCT	ACCGCTTTCCTAATGGTC	fatty acyl-CoA synthetase
Fpfum17	TCTGGAGAAACCTCGAAAGG	ATGCCAATATGCGTGAAATG	longevity assurance factor
Fpfum18	TGATGTGAGGAGCGATGA	TCGAGATTCCTGCCAGCT	longevity assurance factor
Fpfum19	GGAGCCAGATTGGGACAG	ATACCCGAGGAGGAGCAG	ABC transporter
Fpfum21	CGACTGCCAGTATAAAGCC	GTAGCGTAACAGTTTGAGGAG	Zn(II)2Cys6 transcription factor
Fptub	TCGGAAACTCCACCTC	GTCCATACCCTCACCAG	*β*-tubulin
